# Pore Engineering in Carbon Monoliths Through Soft Templating, In Situ Grown Graphene, and Post-Activation for CO_2_ Capture, H_2_ Storage, and Electrochemical Capacitor

**DOI:** 10.3390/nano15120900

**Published:** 2025-06-10

**Authors:** Madhav P. Chavhan, Moomen Marzouki, Mouna Jaouadi, Ouassim Ghodbane, Gabriela Zelenková, Miroslav Almasi, Monika Maříková, Petr Bezdicka, Jakub Tolasz, Natalija Murafa

**Affiliations:** 1 Department of Chemistry, Faculty of Science, University of Ostrava, 30. Dubna 22, 701 03 Ostrava, Czech Republic; gabriela.zelenkova@osu.cz; 2 Institut Préparatoire aux Études des Ingénieurs el Manar (IPEIEM), Campus Universitaire Farhat Hached Tunis, B.P. No. 94, Tunis 1068, Tunisia; moomen.marzouki@ipeiem.utm.tn (M.M.); ing.mouna@gmail.com (M.J.); 3 Laboratoire de Valorisation des Matériaux Utiles, Technopole Borj Cedria, Centre National de Recherches en Sciences des Matériaux (CNRSM), Soliman 8027, Tunisia; 4 Laboratory of Materials, Treatment, and Analysis (LMTA), Biotechpole Sidi Thabet, National Institute of Research and Physico-Chemical Analysis (INRAP), Sidi Thabet 2020, Tunisia; 5 Department of Inorganic Chemistry, Faculty of Science, Pavol Jozef Safarik University, Moyzesova 11, 040 01 Kosice, Slovakia; miroslav.almasi@upjs.sk; 6 Institute of Inorganic Chemistry of the Czech Academy of Sciences, 250 68 Husinec-Řež, Czech Republic; marikova@iic.cas.cz (M.M.); petrb@iic.cas.cz (P.B.); jtolasz@iic.cas.cz (J.T.); murafa@iic.cas.cz (N.M.)

**Keywords:** carbon monoliths, graphene, hierarchical porosity, carbon dioxide capture, hydrogen storage, supercapacitor, sol–gel, carbon xerogel, activation, resorcinol formaldehyde

## Abstract

Controlled porosity with precise pore sizes in carbon monoliths (CMs) is crucial for optimizing performance in electrochemical energy storage and adsorption applications. This study explores the influence of porosity in CMs, developed from polymer precursors via the sol–gel route, employing soft templating, in situ graphene growth, and post-activation. The effects on CO_2_ and H_2_ sorption and electrochemical capacitor (EC) performance are analyzed. Graphene is successfully grown in situ from graphene oxide (GO), as confirmed by several characterization analyses. The amount of GO incorporated influences the crosslink density of the polymer gel, generating various pore structures at both micro- and mesoscales, which impacts performance. For instance, CO_2_ capture peaks at 5.01 mmol g^−1^ (0 °C, 101 kPa) with 10 wt % GO, due to the presence of wider micropores that allow access to ultramicropores. For H_2_ storage, the best performance is achieved with 5 wt % GO, reaching 12.8 mmol g^−1^ (−196 °C, 101 kPa); this is attributed to the enlarged micropore volumes between 0.75 and 2 nm that are accessible by mesopores of 2 to 3 nm. In contrast, for the ECs, lower GO loadings (0.5 to 2 wt %) improve ion accessibility via mesopores (4 to 6 nm), enhancing rate capability through better conduction.

## 1. Introduction

Carbon materials require porosity at different levels of hierarchy for use in adsorption and electrochemical energy storage. Mesopores (2 to 50 nm pore size) and macropores (>50 nm) provide easier transport that promotes rapid diffusion of probe molecules (e.g., H_2_ or CO_2_) or accessibility of solvated electrolyte ions into the interior surface of the carbon structure. On the other hand, micropores (<2 nm) hold most of the surface area, providing active sites for the adsorption of probe molecules or forming a double layer at the interior surface for the storage of electrical energy. Specifically, for H_2_ and CO_2_ molecules, carbon materials containing high micropore features (e.g., surface area or volume) with pore sizes below 1 nm are advantageous, along with the low density and the strong adsorption enthalpy [1,2]. In previous studies on activated carbons and carbide-derived carbons, it was found that H_2_ adsorption measured at −196 °C improved with pore sizes of 0.6–0.7 nm under high pressure and with pore sizes ≥ 0.56 nm at any pressure [1,3,4,5]. For the CO_2_ molecule, the capacity is improved for pore sizes below 0.7 nm at low pressures, and with increasing pressure, the bigger micropores can be filled [5,6]. Furthermore, in the case of double-layer charge storage for electrochemical capacitors (ECs), an optimal match between the pore size and the solvated electrolyte ion size is needed to achieve the best possible performance. The performance depends on many factors, such as the degree of solvation–desolvation, the intercalation of the ions, the swelling and contraction of the carbon layers, and the electrochemical and thermal stability of the electrolyte; thus, finding the exact match is not trivial. In particular, performance in an aqueous electrolyte improves with significant contributions from mesoporosity and greater access to the micropore surface or pore volume in the carbon structure [7]. Furthermore, the electrical conductivity of carbon materials is an additional requirement for EC applications that is responsible for enhanced charge transport, apart from gas adsorption applications.

Graphene, a single layer of carbon atoms arranged in a hexagonal lattice, is renowned for its exceptional properties, such as mechanical strength, thermal and electrical conductivity, optical transparency, and flexibility. Furthermore, a single defect-free graphene sheet has a high theoretical surface area of 2630 m^2^ g^−1^ [8]. However, the restacking of graphene sheets due to van der Waals interactions between adjacent sheets and unavoidable aggregation during the synthesis steps reduces the effective surface area for charge storage, as well as for the adsorption of H_2_ and CO_2_ molecules [8]. Therefore, creating hierarchical porosity in graphene-based materials through activation and/or incorporating graphene into hierarchical porous carbons are considered as promising routes to improve the adsorption capacities of H_2_ and CO_2_ and the electrochemical performance of EC devices [5,9,10,11]. For example, graphene-based carbon with hierarchical porosity developed by physical activation using CO_2_ showed adsorption capacities of 7.74 wt % (1.76 mmol g^−1^) at 0 °C and 1 bar for CO_2_ and 0.75 wt % (3.76 mmol g^−1^) at −196 °C and 1 bar for H_2_ [9]. Furthermore, graphene-based carbon is highly suitable for EC devices due to its excellent electrical conductivity (1 × 10^8^ S m^−1^) [12], which offers a high rate capability. In addition, the incorporation of graphene into hierarchical porous carbon provides a simultaneous improvement in the capacitance, rate capability, and cyclability of electrode material [11].

Carbon monoliths (CMs) offer different shapes and sizes with ease in handling, limited attrition, and non-toxicity compared to the powdered forms of carbon. Most importantly, CMs exhibit remarkable properties, such as a three-dimensional interconnected network with high surface area and specific pore sizes, surface chemistry, electrical conductivity, mechanical strength, and thermal stability, which are highly suitable for use in energy storage, catalysis, gas separation and adsorption, water treatment, and environmental applications [13,14,15,16]. Although there is progress directed toward the development of CMs from lignocellulosic biomass as sustainable sources [13], polymeric precursor-derived carbon is of greater interest due to the continuing dependence on fossil sources [5,16,17]. Customizing porosity at different levels of the pore hierarchy is possible through the sol–gel polycondensation route of hydroxylated benzene (e.g., phenol, cresol, resorcinol, hydroquinone) and aldehyde (e.g., formaldehyde, furfural) [16]. Resorcinol-formaldehyde (RF) is one of the most studied polymer precursors for the production of carbon gel (xerogel/aerogel/cryogel), where micro- and mesoporosity could be easily controlled through processing steps during synthesis (e.g., polymerization rate, drying protocols in solvent removal from wet gel, etc.) and activation protocols [7]. However, CMs still have limitations related to slow diffusion and pressure drop situations. The templating approach is one of the routes in which pores of the mesoscale and macroscale sizes can possibly be created within the CMs to avoid these problems. More specifically, soft templating, typically performed using block copolymers (e.g., P123, F127, or F108), offers simplicity and feasibility in synthesis, and the final monolithic form can be obtained from the entire precursor solution, without macroscopic phase separation during the polymerization stage [16].

With the above discussions, this article aims to induce the desirable pore hierarchy in the CM that suits its use as an electrode for the EC and for the sorption of H_2_ and CO_2_ molecules and to examine its influence on their performance. For this purpose, the numbers of CMs were developed from the sol–gel polymerization route of the RF precursor, followed by carbonization with the following effective modifications: (i) soft templating combination using the addition of Pluronic F127 as a surfactant in the precursor solution to induce mesoscale pores for rapid diffusion (or transport) of electrolyte ions or gas probe molecules; (ii) in situ growth of graphene from the addition of different amounts of graphene oxide (GO) in the precursor solution to tune micro- and mesoporosity; and (iii) post-physical activation using CO_2_ exposure after the end of the carbonization protocol to enhance microfeatures for effective charge storage and sorption of gas molecules. The advantages of the CM developed in this study are that (a) it can be obtained from the entire initial precursor solution (i.e., RF sol + GO + F127) without macroscopic phase separation during sol–gel polycondensation and that (b) it is a possible methodology for the simultaneous growth of graphene in situ and removal of the soft template in the carbonization protocol, followed by post-CO_2_ activation.

## 2. Experimental Section

### 2.1. Synthesis of CM and In Situ Incorporation of Graphene

The detailed synthetic procedure to make graphene oxide (GO) from graphite (<20 µm, Sigma-Aldrich, St. Louis, MO, USA) is described in our previous study [5]. For CM synthesis, first, 5.5 g of resorcinol (min. 98%, Mach Chemikalie, Slezská Ostrava, Czech Republic) was dissolved in a solution of 8.1 g of formaldehyde (38% A.G., stabilized with methanol, Mach Chemikalie) under magnetic stirring for 20 min. Next, this solution was poured into the prior made solution from 1 h of stirring with 5.5 g of Pluronic F127 (bio reagent, Sigma-Aldrich), 17.5 g of ethanol (96% A.G., Mach Chemikalie), and 17.5 g of deionized (DI) water containing different weight percentages (wt %) of dispersed GO and stirred for the next 10 min. The weight percentages (wt %) of GO were taken as 0.5%, 2%, 5%, and 10% in DI water. Subsequently, 2.6 mL of aqueous solution of 1 wt % sodium carbonate (Na_2_CO_3_, Lachema Chemapol, Neratovice, Czech Republic) was added as a catalyst. The resulting solutions were then poured into plastic containers (7 cm in height, 3 cm in diameter), sealed, and kept for polymerization at 70 °C for 24 h. For 0 wt % of GO (i.e., no addition of GO in DI water), a successful gel formation occurred. However, the addition of GO hinders the gelation process, and therefore the pH of the resulting solutions from GO addition was adjusted to 8 by adding more catalyst [5]. Finally, all the wet gels were removed from the cylinders, cut into pieces with the same diameter, and dried at 70 °C for another 24 h. The pyrolysis was then carried out in a tubular furnace with an inert flow of N_2_ (0.2 L min^−1^). The pyrolysis protocol consisted of heating from room temperature to 100 °C at 10 °C min^−1^ with an isothermal step for 30 min, followed by heating to 400 °C at 1 °C min^−1^ with an isothermal step for 1 h, and then heating to 500 °C at 2 °C min^−1^ with an isothermal step for 2 h to ensure complete decomposition of the F127 template. Furthermore, the re-pyrolysis protocol was carried out on these monolithic samples by heating from room temperature to 100 °C (10 °C min^−1^) with an isothermal step of 30 min and then heating to 900 °C (5 °C min^−1^) with an isothermal step for 1 h. CO_2_ activation was immediately started after the end of the re-pyrolysis protocol by switching the flow from N_2_ to CO_2_ with a flow rate of 0.1 L min^−1^ with an isothermal step for 3 h at 900 °C. After the end of CO_2_ activation, the flow of N_2_ was restored to cool the furnace to room temperature. Finally, the CM samples obtained were designated as CM-xGO, where x represents a weight percentage of GO in the precursor solution, and without GO it is simply represented as ‘CM’.

### 2.2. Characterization of Samples

The elemental composition (content of C, H, N and S in wt %) of the synthesized samples was determined using the Vario MICRO CHNS elemental analyzer from Elementar Analysensysteme GmbH (Langenselbold, Germany). The quantitative digestion of the samples occurs by combustion at 1800 °C, and the separation of the resulting gaseous components occurs using a temperature-programmed desorption column for separation without overlapping peaks. The oxygen content in the sample is calculated by subtracting the amounts of carbon, hydrogen, nitrogen, and sulfur from 100%.

The detection of surface functional groups within the samples was performed using a Nicolet 6700 Fourier transform infrared (FTIR) spectrometer connected to a single reflection diamond attenuated total reflection (ATR) crystal. The spectra were acquired from 128 scans at a resolution of 4 cm^−1^ within the wavenumber range of 400 to 4000 cm^−1^.

For X-ray diffraction (XRD) measurements, the samples were adjusted onto Si zero background sample holders. Diffraction patterns were collected with a Malvern PANalytical Empyrean, series 3 diffractometer equipped with a conventional X-ray tube (Co_Kα_ radiation, 40 kV, 30 mA, line focus, *λ* = 1.78901 Å, 1.7929 Å, respectively), multicore optics, and a linear position-sensitive detector PIXCel3D detector. In this case, we used conventional Bragg–Brentano geometry with the iCore optical module set with a 0.03 rad Soller slit, a 0.5° divergence slit, and a 14 mm mask in the incident beam. The dCore optical module set with a 0.5° anti-scatter slit and a 0.04 rad Soller slit was used in the diffracted beam. X-ray patterns were collected in the 2 theta range of 5 to 90° with a step of 0.013° and 500 s per step, producing a scan of about 3 h 40 min. XRD patterns were not pretreated before interpretation, as no background correction was needed. The structural and lattice parameters were calculated from the XRD data using following Equations (1)–(4) [18,19].

(1) $$d_{002}=\frac{\lambda }{2sin\theta_{002}}$$

(2) $$L_{c}=\frac{K_{c}\lambda }{\beta_{002}cos\theta_{002}}$$

(3) $$L_{a}=\frac{K_{a}\lambda }{\beta_{100}cos\theta_{100}}$$

(4) $$N_{av}=\frac{L_{c}}{d_{002}}+1$$

where $d_{002}$ is the inter-layer spacing, $L_{c}$ is the crystallite height, $L_{a}$ is the crystallite diameter, $N_{av}$ is the average number of aromatic layers per carbon crystallite, λ is the wavelength of the incident X-ray, $\theta_{002}$ and $\theta_{100}$ are the positions of the (0 0 2) and (1 0 0) planes in degrees, respectively, $\beta_{002}$ and $\beta_{100}$ are the full width at half maximum (FWHM) of the (0 0 2) and (1 0 0) planes, respectively, and $K_{c}=0.89$ and $K_{a}=1.84$ [19,20].

Raman spectra were acquired using a DXR Raman microscope (Thermo Scientific, Waltham, MA, USA); 256 two-second scans were accumulated with the laser 532 nm (1 mW), 50 μm slit under the 10× objective of the Olympus microscope in full range.

A NovaNanoSEM 450 scanning electron microscope (SEM) was used (FEI, Brno, Czech Republic) to observe the agglomerates of the samples with a choice of accelerating voltage in the range of 1 to 30 kV using the basic Everhart–Thornley detector (ETD) and the high-resolution through-the-lens detector (TLD) for secondary electrons. The surface of the measured samples was cleaned with the UV-prep for the SEM device (2 spi, USA). The samples were first mixed in water to form a fine suspension. Then, 10 µL of this suspension was dropped onto a polished silicon chip and dried under laboratory conditions. Before actual measurement, the silicon chip with the sample was cleaned using the UV-prep device. In the UV-prep chamber, the pressure was reduced to 56 kPa (420 torr), and then the sample was irradiated with UV radiation for 2 min. This cleaning, from residual contamination from the air, ensured a better image (lower noise) in high-resolution observation and reduced contamination of the sample under the electron beam. All samples were measured with an accelerating voltage of 5 kV using a secondary electron detector in immersion lens mode for high resolution.

The crystalline structure, shape, and size of the porous CMs were measured by high-resolution transmission electron microscopy (HRTEM) using a Talos F200X TEM microscope (FEI, Czech Republic) with an acceleration voltage of 200 kV. Standard copper grids covered with a thin transparent holey carbon film for high-resolution observation of the samples were used for sample preparation for TEM. The fine suspension was placed in an ultrasonic bath for a few minutes and then dropped (5 µL) onto a copper grid. The samples were dried freely at room temperature.

### 2.3. Gas Physisorption Experiments

The sorption tests using various gas probe molecules (N_2_, H_2_, and CO_2_) were conducted on the Autosorb iQ-XR manometric system manufactured by Quantachrome instruments, Boynton Beach, FL, USA. Before conducting sorption studies, all the samples were subjected to degassing at a temperature of 300 °C under vacuum. The degassing process involved heating the samples at a rate of 3 °C min^−1^ and allowing them to soak for approximately 18 h at the final temperature. The sorption of N_2_ and H_2_ was carried out at a temperature of −196 °C, while the sorption of CO_2_ was measured at a temperature of 0 °C. The acquired isotherms were analyzed using the ASiQwin program, which is integrated into Quantachrome devices. The micropore characteristics, including pore volume (*V_mic_*) and area (*S_mic_*), as well as the mesopore characteristics, including pore volume (*V_meso_*) and area (*S_meso_*), were determined using quenched solid density functional theory (QSDFT) applied to nitrogen adsorption isotherms. The QSDFT adsorption kernel assumes that the pores are either slit-shaped with a pore width less than 2 nm, or cylindrical-shaped with a pore width greater than 2 nm. This assumption was made to generate pore size distribution plots. Additionally, the Brunauer–Emmett–Teller multipoint method was applied to nitrogen adsorption isotherms to estimate the BET area. Similarly, the pore volumes and areas of the micropores were determined by evaluating the micropore characteristics using CO_2_ adsorption isotherms. This evaluation was conducted using the grand canonical Monte Carlo kernel, assuming that the micropores had a slit-shaped structure. Furthermore, ultramicropore features (≤0.72 nm pore sizes) represented by the cumulative pore volume, *V_umic_*, and cumulative area, *S_umic_*, were assessed separately from these CO_2_ adsorption isotherms. The storage capacity for CO_2_ and H_2_ was determined by measuring the amount of each probe molecule adsorbed at a pressure of 1 bar. These capacities were expressed in units of ‘cm^3^(STP) g^−1^’ and ‘mmol g^−1^’ and in weight percentage as ‘wt %’.

### 2.4. Electrochemical Characterization

The preliminary examinations of the CM samples were carried out in a three-electrode cell utilizing glassy carbon as the working electrode with a geometric surface area of 0.07 cm^2^, a platinum electrode as the counter electrode, and an Ag/AgCl electrode as the reference electrode, with 2 M aqueous KOH serving as the electrolyte. To create coatings on a glassy carbon electrode, each CM sample was disseminated in a 5 wt % Nafion solution (Sigma Aldrich) that contained ethanol solvent (96% Penta Chemicals, Prague, Czech Republic) using ultrasonification. Subsequently, a volume of 10 µL of the dispersed solution was transferred onto a polished glassy surface using a pipette. The electrode was then subjected to additional drying through evaporation under normal atmospheric circumstances. The EcaFlow potentiostat/galvanostat analyzer from Istran was used to record the cyclic voltammetry (CV) plots of each of the prepared electrodes.

For the electrochemical characterization performed in a two-electrode configuration, the working electrodes are composed of active material, carbon black, and polytetrafluoroethylene (PTFE 60% in water) in a mass ratio of 75:15:10 mixed in ethanol. The resulting film paste is then pressed onto a nickel foam, used as a current collector, at a pressure of 10 MPa and dried at 105 °C for 16 h in the oven. The symmetric device is assembled with a Swagelok cell by sandwiching the filter paper impregnated with KOH electrolyte, which is used as a separator between electrodes. The mass and surface area of the film paste are equal to 2 mg and 0.2 cm^2^, respectively, and the mass of the nickel foam is 12 mg. The electrochemical measurements are carried out at room temperature with a Biologic potentiostat/galvanostat system in 3 M KOH aqueous electrolyte prepared with deionized water. CV curves are realized between 0 and 0.9 V at scan rates varying in the range of 5 to 20 mV s^−1^. Galvanostatic charge−discharge (GCD) curves are realized at various current densities. The specific capacitance ($C_{s}$) values are calculated from the discharge curves recorded in the two-electrode setup by the following Equation (5):

(5) $$C_{s}=\frac{4\;I\;\Delta t}{m\;\Delta E}$$

where I (A) is the applied current, ΔE (V) is the applied voltage, Δt (s) is the discharge time, and m (g) is the total mass of the active material on both electrodes.

## 3. Results and Discussions

### 3.1. Formation of CM with Compositional and Structural Changes

It is well known that the mechanism of the crosslinking of the RF gel involves an addition reaction to generate hydroxymethyl derivatives (–CH_2_OH), followed by a condensation reaction to form a gel network connected with methylene (–CH_2_–) and methylene ether (-CH_2_OCH_2_-) [16]. The addition of GO, which contains a large number of hydrophilic oxygen groups, as confirmed from the FTIR spectra (Figure S1) of graphite oxide as a precursor of GO, improves miscibility with the RF solution and forms connections between the hydroxymethyl derivatives. For example, the esterification reaction occurs between the –CH_2_OH intermediates and the –COOH groups from GO [5,21]. Furthermore, the added F127 template interacts with these oligomers through hydrogen bonding, which forms micelles [16,22]. The expansion of micelles occurs during the polymerization process and starts to cluster in flocs. Growth begins with the formation of a co-continuous structure whose size continues to increase with the progress of polymerization, while an increase in the viscosity of the sol can be observed. Finally, during the transition from sol to gel, the growth of these domains stops, and gelling occurs throughout the entire volume of sol [16]. Thus, the monolithic gel formed is composed of the crosslinked polymerized phase that traps the solvent (water + ethanol) within. The removal of solvent from the interior of the gel network occurs during the drying stage, leading to a lightweight, porous organic monolithic gel. Finally, the CM is obtained by decomposition of the F127 template and the reduction of GO to graphene during the carbonization protocol and after activation of pores by exposure to CO_2_. The role of ethanol in conjunction with water as a solvent is essential to avoid macroscopic phase separation during polymerization [16]. However, the cloudy nature of all gels formed after polymerization indicates the unavoidable phase separation at the micrometric scale. Furthermore, we chose the lesser amount of GO (below 10 wt %) in order to avoid such phase separation at the macroscopic level, as well as to prevent future agglomerations of graphene grown in situ. In addition, a low heating rate, as well as more holding time to attain temperatures of 400 °C and 500 °C, was chosen during the pyrolysis protocol to complete the degradation of the F127 template and to avoid more shrinkage or collapse of pores in the monolithic form [5,16]. Additionally, the high carbonization temperature (900 °C) was chosen based on information that showed the complete reduction of GO to graphene, as previously reported [5]. Pictures of the monolithic samples prepared are presented in Figure S2.

Table 1 presents the elemental composition of the CM samples. A clear trend of decrease in the % of C and increase in the % of O is observed with the amount of GO addition in the precursor solution. The additional oxygen induced in the CM samples comes mainly from the oxygen available in GO used in different proportions that interact with the hydroxymethyl derivatives during the initial polymerization stage, as the period of exposure to CO_2_ during activation was the same for all samples. It is obvious that the oxidation of CMs during activation must lead to a decrease in the content of elemental carbon, but this is a similar level in all of these samples. Furthermore, since CO_2_ activation was carried out at a high temperature (900 °C), there is only carbon oxidation that involves pore etching and no introduction of surface oxygen functionalities into the carbonaceous skeleton. This is further supported by FTIR spectroscopy analyses of the final carbonaceous samples (Figure S3). Thus, the oxygen remaining in the CM skeleton is mainly from the reduction of GO, and its proportion increases with the amount of GO loading in the precursor solution recipe.

To understand the structural changes that emerged in the carbonaceous matrix from template decomposition, graphene growth in situ, and CO_2_ activation, XRD patterns were obtained (Figure S4). As usual, two broad diffusion maxima, ascribed to the planes (0 0 2) and (1 0 0), are observed that are related to the ordering of the aromatic structure and the degree of condensation of the aromatic ring appearing in the CM samples, respectively [16,17]. The detailed structural and lattice parameters calculated from the XRD analyses are further summarized in Table 1. Essentially, the values of the crystallite height ($L_{c}$) and average number of aromatic layers per carbon crystallite ($N_{av}$) are comparable, indicating that the GO addition in the precursor sol does not affect the ordering of the aromatic structures in these CM samples. Similarly, the values of the interlayer spacing ($d_{002}$) suggest no change in the number of graphitic layers organized within the structures of all the CM samples. Furthermore, a small change in the crystallite diameter ($L_{a}$) values are observed in these samples, which are related to the crystallinity [17]. However, such a lower content of graphene [5] and its effect on structural changes in the carbon matrix are not clearly detectable by XRD and, therefore, need further investigation.

**Figure 1:**
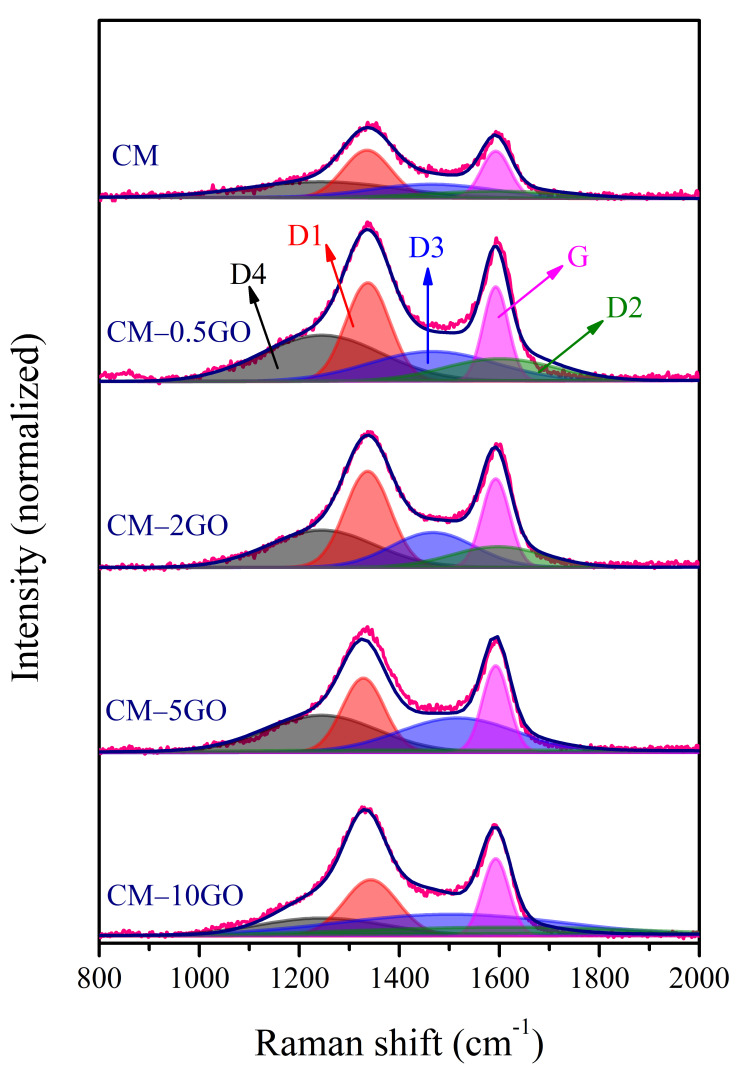
Fitting of Raman spectra for CM samples.

The insight into the structural changes and the degree of crystallinity of the CM samples was further understood from the Raman spectroscopy analyses. Two characteristic peaks around 1337 ± 5 cm^−1^ (D band) and ~1591 cm^−1^ (G band) appear in the CM samples (Figure S5), where the former band represents a disordered carbon structure and the defects such as vacancies, functional groups, oxidation-induced defects, or the presence of charge impurities, and the latter band originates from sp^2^ carbon [23]. The rough estimate of the degree of graphitization is usually determined from the intensity ratios of the D band to the G band (*I_D_/I_G_*), and the calculated values are noted as 1.1, 1.06, 1.05, 1.07, and 1.13 for the CM, CM-0.5GO, CM-2GO, CM-5GO, and CM-10GO samples, respectively. However, from such a rough estimate with smaller changes in these values, one cannot predict the changes in carbon structure that arose from the growth of graphene in situ and the removal of the template during the carbonization protocol and further post-activation. Therefore, a detailed analysis was carried out by fitting the first-order region of Raman spectra into five bands, referred to as D4, D1, D3, G, and D2 (Figure 1), with fixed positions of the D4 and G bands, according to the procedure reported in the literature [18,24]. The D1 band represents the graphitic lattice vibration mode with A_1g_ symmetry and is assigned to the presence of heteroatoms or defects [25,26]. The D2 band is assigned to the stretching mode of the aromatic layers involving graphene layers that are not directly inserted between two other graphene layers [18,27]. The D3 band generally arises as a very broad band that is assigned to the amorphous sp^2^-bonded forms generated from organic molecules, fragments, or functional groups. Finally, the D4 band is ascribed to the poorly organized molecules, such as coal chars or soot [28,29,30], and is also attributed to the sp^3^-sp^2^ mixed sites at the periphery of crystallites [31]. The fitting results from the Raman spectra are listed in Table 2. The decrease in the full width at half maximum (FWHM) values of the G band suggests an improvement in the structural order of the CM samples obtained with the addition of GO to the precursor solution. These FWHM values of the G bands obtained in this study are lower than those reported with petroleum coke-derived carbon [30] and carbon xerogel obtained from an RF precursor [18], indicating a greater degree of organization in CM samples developed from graphene grown in situ. The presence of the D1 band is related to the vibration mode of the graphitic lattice due to defects and the presence of oxygen as heteroatoms, and its decrease in peak area confirms the diminishing defects in CM samples with the increase in graphene proportions. Furthermore, the D2 band appears because of the presence of the D1 band, and its relative decrease in intensity (i.e., fitted peak area) for CM samples obtained with the addition of GO from 0.5 wt % to 10 wt % is another measure of the increase in organization degree with higher graphene content. Furthermore, there are several studies in which lower FWHM values of the D1 and G bands appear in carbon samples, indicating an increased order of degree of graphitization [25,32], such as in the analyses carried out in this study.

### 3.2. Morphological Changes and Porosity Creation

**Figure 2:**
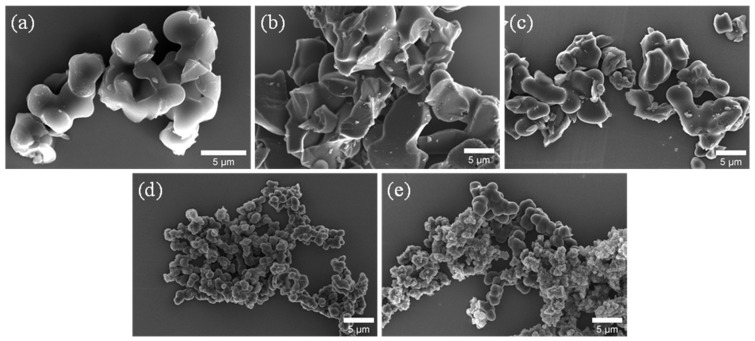
SEM images of (**a**) CM, (**b**) CM-0.5GO, (**c**) CM-2GO, (**d**) CM-5GO, and (**e**) CM-10GO.

**Figure 3:**
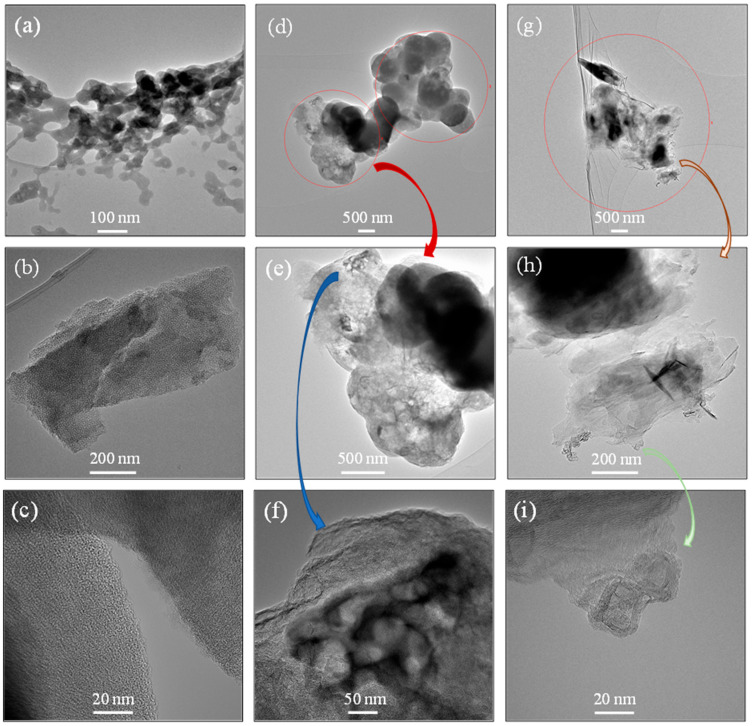
TEM images of (**a**,**b**) CM, (**c**) CM-10GO, and (**d**–**i**) CM-5GO.

The morphological changes that arise in CM samples are well understood from the SEM (Figure 2) and TEM (Figure 3) images. Figure 2a shows the interconnected carbonaceous skeleton network formed after the drying and pyrolysis protocols applied to a monolithic gel where the crosslinked network of clusters developed through bonding between the RF oligomers and the F127 template. The size of the interconnected carbon nanoparticulates is obviously larger compared to the carbonaceous network developed through RF crosslinking alone [17], because of the growth of micelles (developed from F127 and RF oligomers) during polymerization. With the addition of GO, more connections are formed during the polymerization stage between the hydroxymethyl derivatives and the hydrophilic oxygen groups of GO. Thus, with increasing concentrations of GO, the interconnected network clusters start to develop rapidly through a smaller space during polymerization. This led to a more interconnected network, which formed in the CM samples with increasing graphene content, as observed in the SEM images in Figure 2b–e. In addition, the growth of the co-continuous structure of RF oligomers + GO formed with the template during polymerization is restricted in comparison to polymerization with no added GO suspension. Thus, the sizes of the carbon nanoparticulates with higher graphene content (with 5% and 10% GO) are smaller compared to the CM sample obtained without the presence of graphene (see Figure 2d,e). Furthermore, the porosity at the macroscale formed between the interconnected carbon nanoparticulate network can be approximately visualized in all these SEM images. The interconnected network of the carbonaceous skeleton is also observed in the TEM images for the CM samples (Figure 3a and Figure S6). The presence of the mesopores and macropores that arose in the CM samples is clearly visible in these images. Further insight into the pores at the lower level of the mesoscale can be derived from the TEM images shown in Figure 3b,c, where the mesopores are randomly arranged with a somewhat wormlike geometry. Furthermore, the polymerization mechanism between the hydrophilic oxygen groups of GO and the hydroxymethyl derivatives is confirmed from the interconnected network formed in the CM samples, as observed in the TEM images in Figure 3d,e, where graphene was successfully incorporated into the carbonaceous skeleton. In addition, clear visual evidence of the presence of graphene incorporation can be seen in Figure 3f,g. Finally, the graphitic domains are also visualized in the TEM image of Figure 3i, where the layered structure of the graphitic cores is observed.

**Figure 4:**
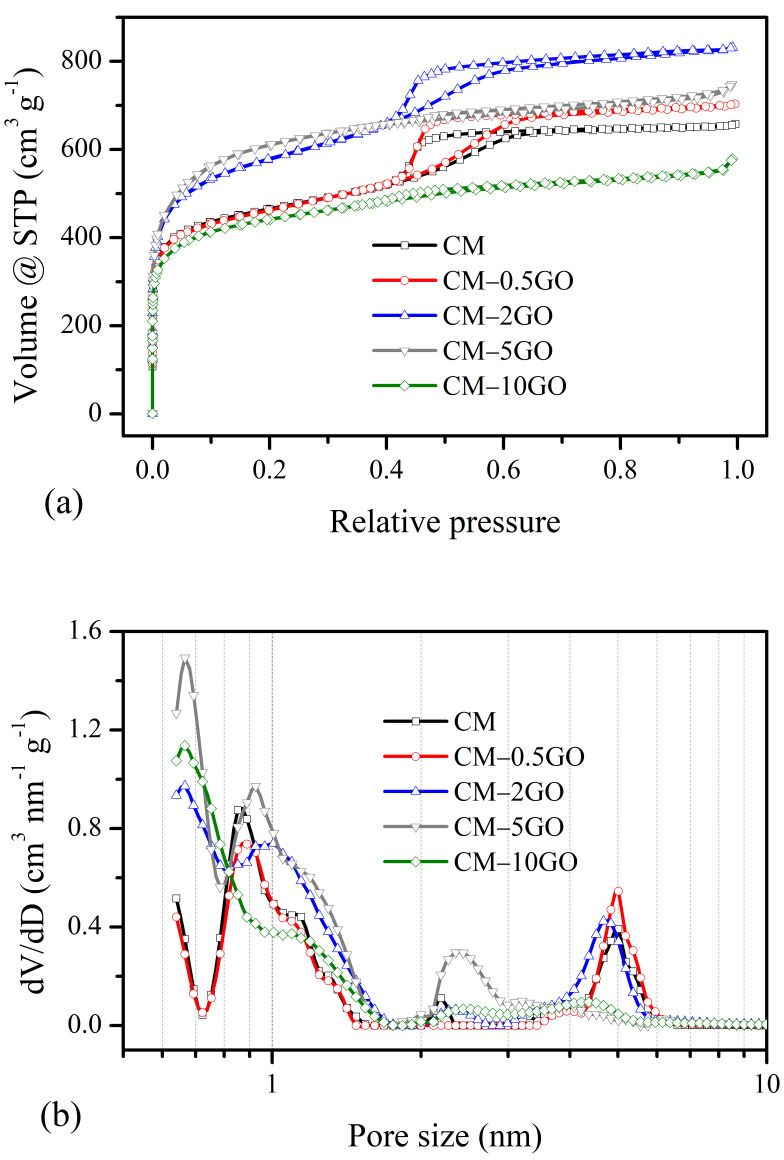
(**a**) Nitrogen adsorption–desorption isotherms at −196 °C and corresponding (**b**) pore size distributions using QSDFT equilibrium model.

Detailed information on the porous texture developed in the CM samples was further analyzed from the nitrogen adsorption–desorption experiments (Table 3 and Figure 4). The CM derived without graphene and the CM-0.5GO and CM-2GO samples show typical characteristics of a Type IV(a) isotherm (Figure 4a), as classified according to the IUPAC Technical Report 2015 [33]. The presence of hysteresis with this type of isotherm represents pore condensation that usually appears in mesopores wider than ~4 nm. From the QSDFT pore size distribution (Figure 4b), it is further confirmed that the mesopores that appeared in these CM samples have pore sizes of 4 to 6 nm, with a significant contribution of mesopore volume. On the other hand, the CM-5GO and CM-10GO samples show the combination of the Type I(b) and Type IV(b) nature of isotherms. The Type I(b) isotherm represents wider micropores and possibly narrow mesopores (<~2.5 nm), while the latter Type IV(b) isotherm signifies mesopores of smaller width, with the completely reversible nature of the isotherms. This is clearly observed in both samples, with the appearance of wider micropores and mesopore sizes in the 2 to 3 nm range with significant pore volumes, as seen from the pore size distribution plot. The higher uptake in adsorbed volume at a low relative pressure indicates the development of micropore features, and its trend for the CM samples increases with the graphene content up to a 5 wt % addition of GO to the precursor solution (see Table 3). However, with a 10 wt % addition of GO, there is no improvement in micropore features, as observed from the nitrogen sorption experiment, and these features are of the same level as the pure CM sample prepared without the addition of GO. The development of micropore features in CM samples can be understood from polymerization chemistry. With the increased addition of GO, crosslinked network clusters grow in smaller spaces with a time that leads to the predominance of micropores in the CM samples, and with a 10 wt % GO addition, the N_2_ molecule with a kinetic diameter of 0.36 nm [6,34] is probably not fully accessible in narrower micropores. This can be clearly observed from the CO_2_ sorption results, as described in the next section. On the other hand, the mesopore characteristics improve with added GO up to 2 wt %, and then a decrease in the trend of the mesopore characteristics is observed. This means that a rapid network clustering takes place at higher ratios of 5 and 10 wt % of GO leading to a loss in mesopore characteristics. The evidence of rapid clustering also leads to restricted growth of network clusters with smaller carbon nanoparticulate sizes, which is already confirmed in the SEM images of the CM-5GO and CM-10GO samples, as seen before in Figure 2d,e. The BET area is also presented in Table 3 to obtain an understanding of the overall contribution of the micropore features to the surface area, which is one of the important characteristics responsible for the enhanced performance of the CM samples in the applications considered in this study.

### 3.3. CO_2_ and H_2_ Sorption

**Figure 5:**
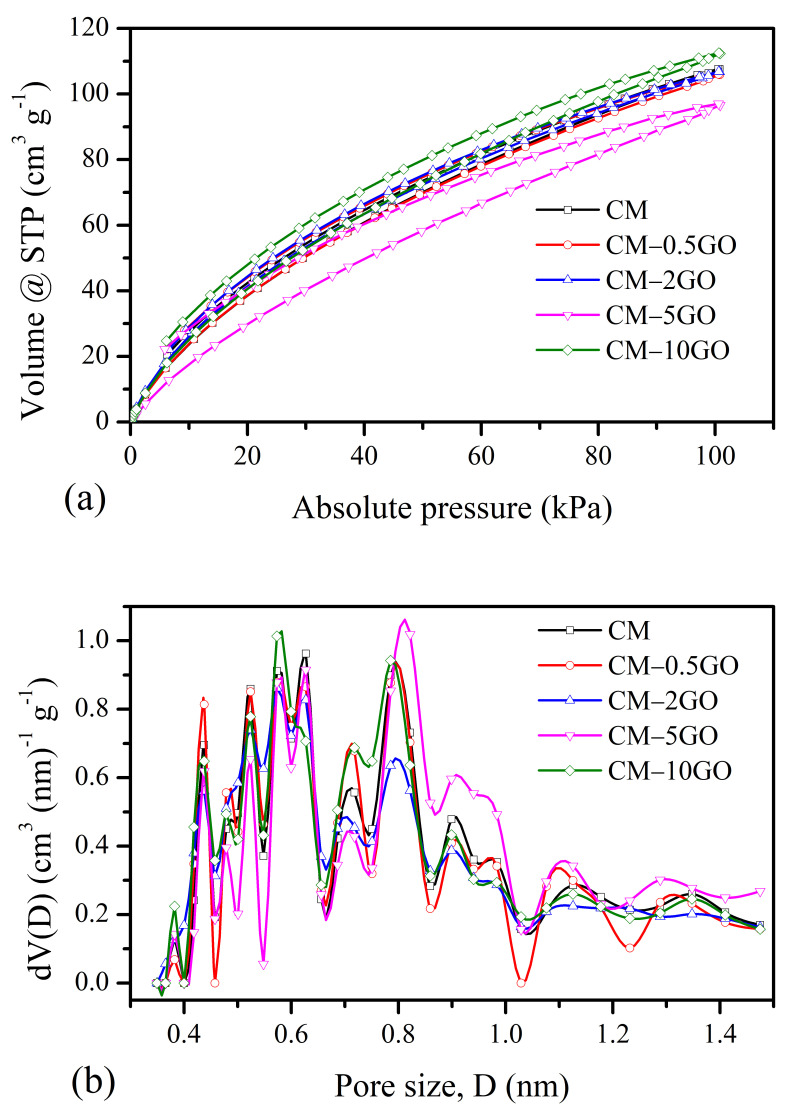
(**a**) Carbon dioxide adsorption–desorption isotherms at 0 °C and corresponding (**b**) pore size distributions using Monte Carlo model.

The CO_2_ adsorption storage capacities of the CM samples measured at 0 °C are tabulated in Table 4. These storage capacities in different units are evaluated from adsorption isotherms (Figure 5a) measured up to 101 kPa. As mentioned before, the adsorption capacity of CO_2_ depends on micropore characteristics. A comparison of the CM samples shows that the highest capacity is found for the CM-10GO samples, as seen from the isotherms and Table 4, with a value of 5.01 mmol g^−1^ (=112.3 cm^3^(STP) g^−1^, 22.05 wt %). However, porosity analyses from the nitrogen sorption experiments (see Table 3) show that the CM-10GO sample has lower micropore characteristics compared to the CM-2GO and CM-5GO samples, as observed from the BET area and the QSDFT pore volume and surface area. One of the reasons behind the high performance of the CM-10GO sample is the rapid network clusters developed over a smaller space with time, leading to smaller-sized micropores (also called ultramicropores) that provide large sites for CO_2_ adsorption, which are inaccessible for the N_2_ molecule (0.36 nm diameter). This can be understood from the pore size distribution developed by applying the Monte Carlo model to the CO_2_ adsorption data, as presented in Figure 5b, together with the micropore characteristics values tabulated in Table 4. Therefore, wider micropores (as confirmed by the Type I(b) nitrogen isotherm) with significant volume, which provides accessibility for the CO_2_ molecule (0.33 nm diameter) towards smaller-sized (ultra) micropores (as confirmed by the pore volume (or surface area) below 1.47 nm) in the CM-10GO sample, are the reason for the higher CO_2_ adsorption capacity. More specifically, the significant pore volume below 0.72 nm (i.e., ultramicropores) that appeared in the CM-10GO sample, as seen in Figure 5b and Table 4, is the reason for their higher adsorption affinity, which occupies the CO_2_ molecule that has a diameter size of 0.33 nm [35]. This observation can be made based on the results of the CM-5GO and CM-10GO samples by comparing their pore size distributions in Figure 5b and their ultramicropore features in Table 4. Furthermore, the high content of oxygen present in the skeleton of the CM-10GO sample provides more affinity for CO_2_ adsorption due to strong polar interaction [8,35], as confirmed by the similar level of ultramicroporosity features developed in the CM-2GO sample. Thus, the combined effect of microporous features as well as the presence of oxygen in the carbon structure is responsible for the high capacity of the CM-10GO sample. This CO_2_ adsorption capacity obtained in this study is further compared with those studies reported in the literature under similar conditions of measurements carried out at 0 °C and 101 kPa [8,35]. For example, KOH-activated porous graphene derived from thermally exfoliated graphite oxide showed a CO_2_ adsorption capacity of 4.06 mmol g^−1^ (17.87 wt %) [8]. GO with 0.2 wt % incorporation in biomass-based carbon foam delivered a capacity of 4.99 mmol g^−1^ [35], which is close to our result. Therefore, based on these results, it can be confirmed that the incorporation of graphene into porous carbon is the best possible way to improve the CO_2_ adsorption capacity of carbon materials.

**Figure 6:**
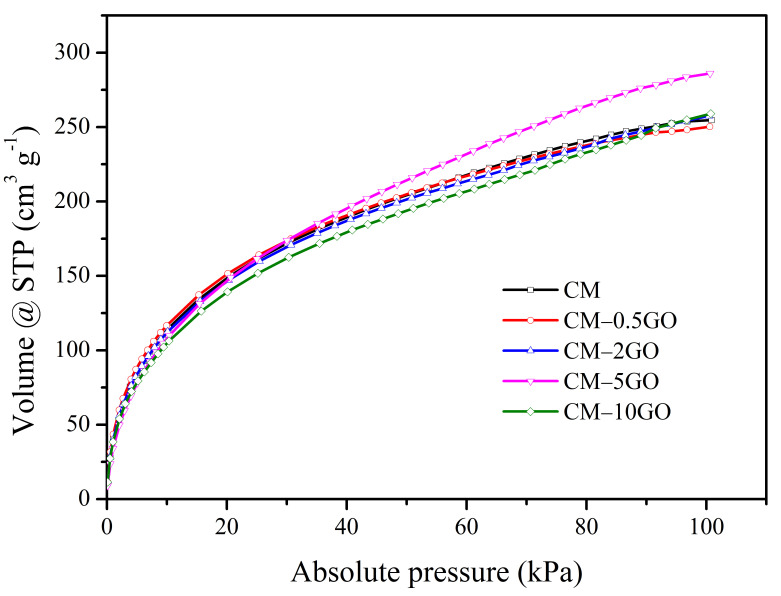
Hydrogen adsorption–desorption isotherms at −196 °C.

Similarly, the H_2_ storage capacities of the CM samples measured up to 101 kPa are summarized in Table 5 from the H_2_ sorption isotherms obtained at −196 °C (Figure 6). However, instead of the CM-10GO sample, the CM-5GO sample shows the highest H_2_ adsorption capacity with a value of 12.8 mmol g^−1^ (=286 cm^3^(STP) g^−1^, 2.6 wt %). All the other samples show a similar level of capacity performance. The large capacity of the CM-5GO sample is directly related to its high micropore volume, as observed in both the N_2_ and CO_2_ sorption data (Table 3 and Table 4). More specifically, a large significant micropore volume in the entire pore size range from 0.75 nm to 2 nm compared to other samples, as observed in both pore size distribution plots (Figure 4b and Figure 5b), is the reason for the high storage of H_2_. Furthermore, the reasonable mesopore volume of a size of 2 to 3 nm may facilitate the diffusion of H_2_ molecules (0.29 nm diameter) into the micropores. Also, note that micropores (measured at low relative pressure) are involved in the estimation of the BET area. However, there is no such relation between H_2_ storage capacity and BET area (Table 3). According to previous studies on carbon materials, pore sizes of 0.6–0.7 nm under high pressure and with pore sizes ≥ 0.56 nm under any pressure are favorable for enhanced hydrogen storage at −196 °C [1,3]. Therefore, the pore size distribution of the CM-5GO sample with a significant volume obtained in this study is also favorable under any pressure for H_2_ storage. Also, it is important to note that the presence of oxygen content has no effect on the H_2_ storage capacity, possibly due to the non-polar nature of the H_2_ molecule, which has low polarizability and interacts weakly with polar surface sites [8]. Furthermore, the comparison of the H_2_ storage capacity obtained in this study is greater than those reported with carbon materials in previous studies under similar measurement conditions (−196 °C and 101 kPa) [1,3,8,23]. For example, KOH-activated hierarchical porous graphene [8] and GO-incorporated ordered mesoporous carbon [23] showed a H_2_ storage capacity of 2.41 wt % (11.88 mmol g^−1^) and 2.3 wt % (11.7 mmol g^−1^), respectively.

### 3.4. Electrochemical Performance

**Figure 7:**
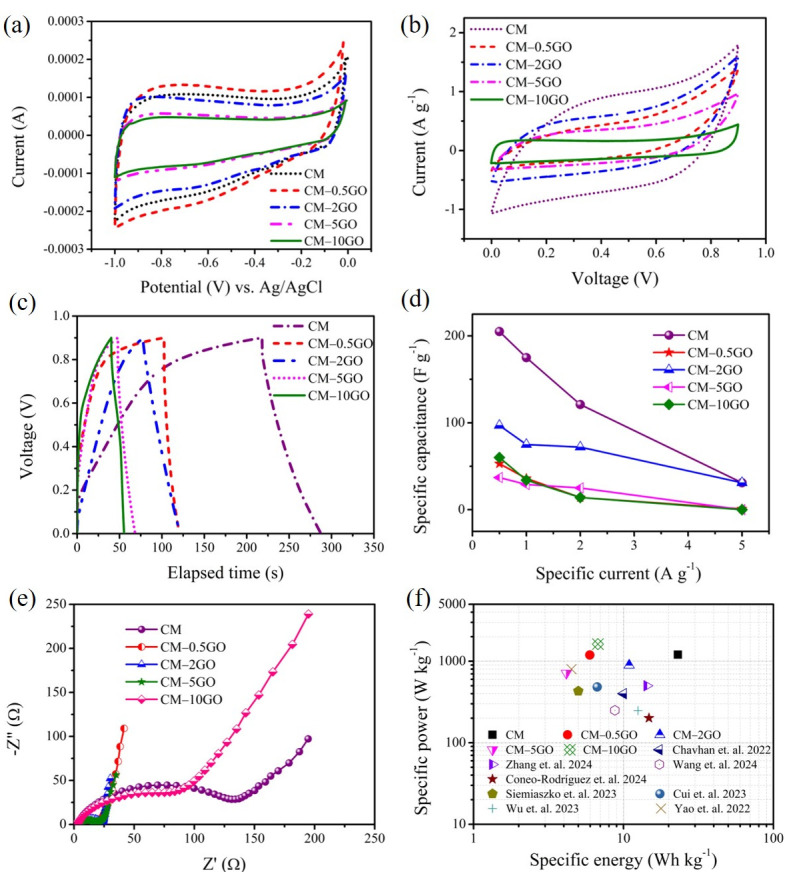
Electrochemical performance of CM samples: (**a**) CV plot in three-electrode cells at 5 mV s^−1^, two-electrode cell result with (**b**) CV plot at 20 mV s^−1^, (**c**) GCD plot at 0.5 A g^−1^, (**d**) rate capability plot, (**e**) Nyquist plot from impedance data, and (**f**) Ragone plot [17,37,38,39,40,41,42,43].

Figure 7 shows the electrochemical performance of the CM samples tested in three and two-electrode cells. The CV experiments carried out within 0 to −1 V in a three-electrode cell (Figure 7a) show quasi-rectangular profiles, typical of capacitive behavior and suggest a charge storage mechanism in the electrochemical double layer. The deviation from an ideal rectangular shape is possibly due to the pore heterogeneity that exists in all types of CM electrodes. Based on areas under CV curves exhibited at 5 mV s^−1^, the electrochemical performance decreases following the order: CM-0.5GO > CM > CM-2GO > CM-5GO > CM-10GO. However, at a higher scan rate of 50 mV s^−1^ (Figure S7), the performance of CM-0.5GO is slightly lower than that of the CM electrode. This result suggests that the pore hierarchy in CM-0.5GO is not favorable at a high scan rate to access all pores for electrolyte ions, while accessibility is facilitated at low scan rates. Furthermore, the tests of the CM samples in a two-electrode setup show a similar nature to the CV results (Figure 7b), with the CM sample (without added GO) showing the highest performance, followed by the CM-2GO sample. The similar nature of the low performance in the CM-0.5GO sample is again in agreement with the CV plots observed at low and high scan rates (Figure 7b and Figure S8). GCD experiments were conducted to validate the results from the CV profiles. The deviations observed in a regular triangular shape in the GCD profiles (Figure 7c), carried out at 0.5 A g^−1^, are in accordance with the mechanism understood from the CV analyses. A similar order of performance is observed in these GCD plots, with the highest performance for CM electrodes, followed by CM-2GO electrodes. From these CV and GCD analyses, the CM, CM-0.5GO, and CM-2GO electrodes show higher performance compared to the CM-5GO and CM-10GO electrodes, possibly due to a significant mesopore volume in the pore sizes of 4 to 6 nm that provides sufficient ion accessibility, while such mesopores are completely absent in the latter types of electrodes. Furthermore, the relative order of performance between the former electrodes, that is, CM, CM-0.5GO, and CM-2GO, is closely related to their respective micropore characteristics (area or volume) determined from the CO_2_ sorption analyses, as seen in Table 4. Furthermore, the role of the enhanced mesopore volume (or area) in the CM-2GO electrode (see Table 3) provides a more stable rate capability performance compared to CMs and other types of electrodes (Figure 7d). However, a significant contribution from the enhanced conduction, as a result of the presence of graphene, which is responsible for the improved rate capability, cannot be ruled out. This is also observed in the Nyquist graph (Figure 7e), where lower impedance values and more capacitive charge storage behavior (as seen from the less slanted lines with respect to the vertical axis) are observed in the graphene-incorporated CM electrodes compared to the pure CM electrode, except for a higher impedance observed in the CM-10GO electrode. The higher impedance values are possibly due to the absence of significant mesopore characteristics in the CM-10GO sample, as seen from the pore size distribution in Figure 4b or the mesopore area or volume values seen in Table 3. Furthermore, it should be noted that these conclusions are based on the use of aqueous KOH solution as an electrolyte, whose solvated ions (K^+^ and OH^-^) have a similar hydrated ionic size of 0.424 nm [36]. The electrochemical results are further compared with those of carbon electrodes derived from the RF precursor in our previous studies [7,17]. Here, the low capacitance values possibly resulted from the induction of more meso- and macroscale voids created in the CM samples that are inefficient in terms of providing rapid electron transfer. Nevertheless, the superior electrochemical behavior of the CM samples can be understood from the Ragone plot (Figure 7f), where these CM electrodes are compared with those recently reported as carbon electrodes using RF precursors under a symmetric two-electrode cell in an aqueous electrolyte (details listed in Table S1) [17,37,38,39,40,41,42,43]. The maximum specific energy of 23.06 Wh kg^−1^ is delivered by the CM electrode at a specific power of 1203 W kg^−1^.

More detailed information on charge transport in CM electrodes is provided by fitting the equivalent electric circuit of the ladder model (Table 6) to the impedance data using ZsimpWin 3.21 software. Here, the selected model represents a branched network of pores with three levels of hierarchy [44]. The convergent values of the equivalent circuit parameters are tabulated in Table 6. It is clearly seen that the charge transfer resistance to access the lower level of pore hierarchy, and the double-layer capacitive charge storage at each respective lower level of pore hierarchy, increases for all of these electrodes. The lower resistance values at the higher level of the pore hierarchy are due to the presence of meso- and macroscale voids created within the interconnected network of all of these CM samples, which provides transport for solvated ions to the lower level of the pore hierarchy. A major difference is observed in the lowest level of pore hierarchy, as seen from the leakage resistance, R_4_, in these electrodes. More specifically, a lower leakage resistance value, as seen in a CM electrode, indicates less branching in the pore networks in the lowest hierarchy [44]. On the other hand, higher values represent more branching in the pore network at the lowest level of pore hierarchy present in all these graphene-incorporated CM electrodes. This can be explained by the polymerization mechanism between the hydrophilic oxygen groups of GO and the hydroxymethyl derivatives, resulting in a more interconnected network, as explained before and seen in the SEM images, confirming the stated hypothesis.

## 4. Conclusions

This article presented the role of porosity, especially the pore hierarchy with specific pore sizes at the micro- and mesoscale developed in CMs from the soft templating, in situ grown graphene, and post-CO_2_ activation, in the performance of CO_2_ and H_2_ sorption and double-layer charge storage in EC. The preparation of CMs reported in this study is advantageous, as it can be obtained from the entire precursor solution without macroscopic phase separation during sol–gel polycondensation and provides a methodology for the simultaneous growth of graphene in situ and removal of the soft template in the pyrolysis protocol, followed by post-CO_2_ activation. This provides simplicity in synthesis as well as an opportunity for bulk production of CMs. The different proportions of GO in the precursor solution induce compositional, structural, and morphological changes in the carbonaceous skeleton of CMs. Furthermore, this affects the rate of polymerization and the crosslink density of the resulting gels, causing various levels of the pore hierarchy to finally be inducible in the micro- and mesopores of the CM samples. The effect of porosity created in these CM samples on the performance of adsorption capacity for CO_2_ and H_2_ and the electrochemical performance for double-layer charge storage in an EC is summarized below.

- For CO_2_ adsorption, the highest adsorption capacity is the result of the enhanced ultramicroporosity, as well as the wider micropores created in the CM-10GO sample. The increased polymerization rate from the addition of a higher concentration of 10% by weight GO loading leads to highly crosslinked network clusters developing over a smaller space, which, upon the use of pyrolysis protocols, leads to those micropore characteristics that are suitable for more CO_2_ adsorption. Furthermore, the high content of oxygen present in the structure of the CM-10GO sample additionally provides affinity for such CO_2_ adsorption.
- In the case of H_2_ storage, the highest significant value of adsorption capacity of 12.8 mmol g^−1^ (at −196 ° C and 101 kPa) is possible in the CM sample, derived from the GO with a loading of 5 wt %. The improved micropore volume in the entire pore sizes from 0.75 nm to 2 nm, as well as the significant accessibility provided by mesopores in the 2 to 3 nm sizes compared to other CM samples, are advantageous in achieving the highest adsorption capacity value for H_2_ storage.
- For CM electrodes, significant access for solvated ions provided by mesopores in the 4 to 6 nm sizes toward enhanced micropores improves the charge storage in the double layer. Such mesopore characteristics are created from the lower amount of GO loadings (0.5 to 2 wt %), where the relatively lower rate of polymerization leads to the formation of larger and fewer network clusters during polymerization compared to high GO loadings, which benefits CMs for the relatively higher values of electrochemical performance. Although more branching in the pore network at the lowest level of the pore hierarchy in graphene-incorporated CM electrodes offers more leakage resistance, the rate capability improves because of enhanced conduction from the incorporation of graphene.

## Figures and Tables

**Table 1:** Elemental composition, and structural and lattice parameters, calculated from XRD data.

Sample	Elemental Composition (%)			Structural and Lattice Parameters Calculated from XRD Analyses			
	C	H	O	$d_{002}$ (A^0^)	$L_{c}$ (A^0^)	$L_{a}$ (A^0^)	$N_{av}$
CM	92.20	1.07	6.70	3.94	12.74	34.81	4.23
CM-0.5GO	90.97	1.00	8.03	4.05	12.34	37.00	4.05
CM-2GO	89.23	0.96	9.67	3.94	12.12	42.80	4.07
CM-5GO	85.49	1.11	13.26	3.89	10.51	47.11	3.70
CM-10GO	82.95	1.63	15.36	3.93	11.19	43.03	3.85

Notations: $d_{002}$ = inter-layer spacing, $L_{c}$ = crystallite height, $L_{a}$ = crystallite diameter, $N_{av}$ = the average number of aromatic layers per carbon crystallite.

**Table 2:** Fitting results of the Raman spectral analysis.

Sample	D4		D1		D3		G		D2	
	Center	FWHM	Center	FWHM	Center	FWHM	Center	FWHM	Center	FWHM
CM	1245	355	1336	112	1465	294	1593	68	1596	333
CM-0.5GO	1245	282	1337	104	1466	297	1593	59	1600	261
CM-2GO	1245	249	1337	107	1467	196	1593	65	1597	218
CM-5GO	1245	263	1328	99	1517	276	1593	63	1597	1137
CM-10GO	1245	301	1343	127	1502	534	1593	62	1611	695

**Table 3:** Porosity data from nitrogen sorption at −196 °C.

Sample	QSDFT Pore Volume (cm^3^ g^−1^)		QSDFT Surface Area (m^2^ g^−1^)		BET area (m^2^ g^−1^)
	*V_mic_*	*V_meso_*	*S_mic_*	*S_meso_*	
CM	0.512	0.434	1483	376	1751
CM-0.5GO	0.496	0.519	1478	408	1727
CM-2GO	0.611	0.583	1448	507	2154
CM-5GO	0.646	0.397	1452	464	2287
CM-10GO	0.518	0.273	1331	257	1661

**Table 4:** CO_2_ adsorption data and microporosity estimation using Monte Carlo model.

Sample	CO_2_ Adsorption (101 kPa, 0 °C)			Micropore (<1.47 nm)		Ultramicropores (≤0.72 nm)	
	cm^3^(STP) g^−1^	mmol g^−1^	wt %	*V_mic_* (cm^3^ g^−1^)	*S_mic_* (m^2^ g^−1^)	*V_umic_* (cm^3^ g^−1^)	*S_umic_* (m^2^ g^−1^)
CM	107.6	4.80	21.13	0.424	1153	0.184	666
CM-0.5GO	105.9	4.72	20.79	0.402	1115	0.189	683
CM-2GO	106.8	4.76	20.96	0.398	1120	0.192	707
CM-5GO	96.6	4.31	18.96	0.44	1108	0.145	515
CM-10GO	112.3	5.01	22.05	0.427	1182	0.194	701

**Table 5:** H_2_ adsorption data.

Sample	H_2_ Adsorption (101 kPa, −196 °C)		
	cm^3^(STP) g^−1^	mmol g^−1^	wt %
CM	255	11.4	2.3
CM-0.5GO	250	11.2	2.3
CM-2GO	258	11.5	2.3
CM-5GO	286	12.8	2.6
CM-10GO	259	11.6	2.3

**Table 6:** Equivalent electric circuit and regressed values of circuit parameters.

Equivalent Electric Circuit (Ladder Model)			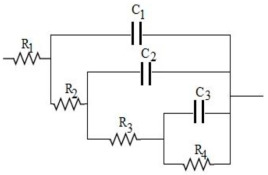					
Type of Electrode	R_1_ (Ω)	C_1_ (F)	R_2_ (Ω)	C_2_ (F)	R_3_ (Ω)	C_3_ (F)	R_4_ (Ω)	Chi^2^
CM	3.8	1.9 × 10^−4^	41	6.4 × 10^−4^	84.1	0.075	125	1.2 × 10^−2^
CM-0.5GO	3.2	2.0 × 10^−4^	14	0.019	11.6	0.110	486	1.4 × 10^−2^
CM-2GO	3.2	2.2 × 10^−4^	12	0.003	7.9	0.279	307	4.7 × 10^−2^
CM-5GO	4.3	1.3 × 10^−4^	12	0.014	9.2	0.184	196	2.5 × 10^−2^
CM-10GO	3.4	1.2 × 10^−4^	36	5.8 × 10^−4^	78	0.014	1231	4.1 × 10^−2^

Notations: R_1_: circuit equivalent series resistance; C_1_: capacitance in first level of pore hierarchy; C_2_ and C_3_: capacitances for the intermediate and the lowest hierarchy pores, respectively; R_2_ and R_3_: resistances to charge propagation at the intermediate and the lowest hierarchal pores, respectively; R_4_: leakage resistance associated with C_3_; Chi^2^: goodness of fit.

## Data Availability

Data will be made available on request.

## Supplementary Materials

The following supporting information can be downloaded at https://www.mdpi.com/article/10.3390/nano15120900/s1, Figure S1: FTIR spectra of graphite oxide; Figure S2: Pictures of prepared CM samples; Figure S3: FTIR spectra of CM samples; Figure S4: XRD spectra of CM samples; Figure S5: Raman spectra of CM samples; Figure S6: TEM image of CM-2GO; Figure S7: CV plot in a three-electrode cell at 50 mV s^−1^; Figure S8: CV plot in a two-electrode cell at 5 mV s^−1^; Table S1: Comparison of the electrochemical performance of CM electrodes with recently reported carbon electrodes from resorcinol-formaldehyde precursors under a two-electrode symmetric cell in an aqueous electrolyte.
