# Supplementary material for: Pore Engineering in Carbon Monoliths Through Soft Templating, In Situ Grown Graphene, and Post-Activation for CO2 Capture, H2 Storage, and Electrochemical Capacitor
Source: Nanomaterials (Basel). 2025 Jun 10;15(12):900. doi: 10.3390/nano15120900 (PMC12196035; doi:10.3390/nano15120900)
Supplement: Supplementary file 1 [file nanomaterials-15-00900-s001.zip › nanomaterials-3642490-supplementary.pdf]

Supporting information

Pore engineering in carbon monoliths through soft templating, *in situ* grown graphene, and post-activation for CO_2 capture, H_2 storage, and electrochemical capacitor

Madhav P. Chavhan [1,*], Moomen Marzouki [2], Mouna Jaouadi [2,3], Ouassim Ghodbane [4,*], Gabriela Zelenková [1], Miroslav Almasi [5], Monika Maříková [6], Petr Bezdicka [6], Jakub Tolasz [6], Natalija Murafa [6]

[1] Faculty of Science, University of Ostrava, 30. dubna 22, Ostrava 701 03, Czech Republic

[2] Institut préparatoire aux études des ingénieurs el Manar (IPEIEM), campus universitaire Farhat Hached Tunis, B.P.no.94, Romamana 1068, Tunisia.

[3] Laboratoire de Valorisation des Matériaux Utiles, Technopole Borj Cedria, Centre National de Recherches en Sciences des Matériaux (CNRSM), Soliman, Tunisia

[4] Laboratory of Materials, Treatment, and Analysis (LMTA), Biotechpole Sidi Thabet, National Institute of Research and Physico-Chemical Analysis (INRAP), 2020, Sidi Thabet, Tunisia

[5] Department of Inorganic Chemistry, Faculty of Science, Pavol Jozef Safarik University, Moyzesova 11, 040 01 Kosice, Slovak Republic

[6] Institute of Inorganic Chemistry of the Czech Academy of Sciences, 250 68 Husinec – Řež, Czech Republic

Corresponding authors[*]

(Madhav.Chavhan@osu.cz) Madhav P. Chavhan

(ouassim.ghodbane@inrap.rnrt.tn) Ouassim Ghodbane

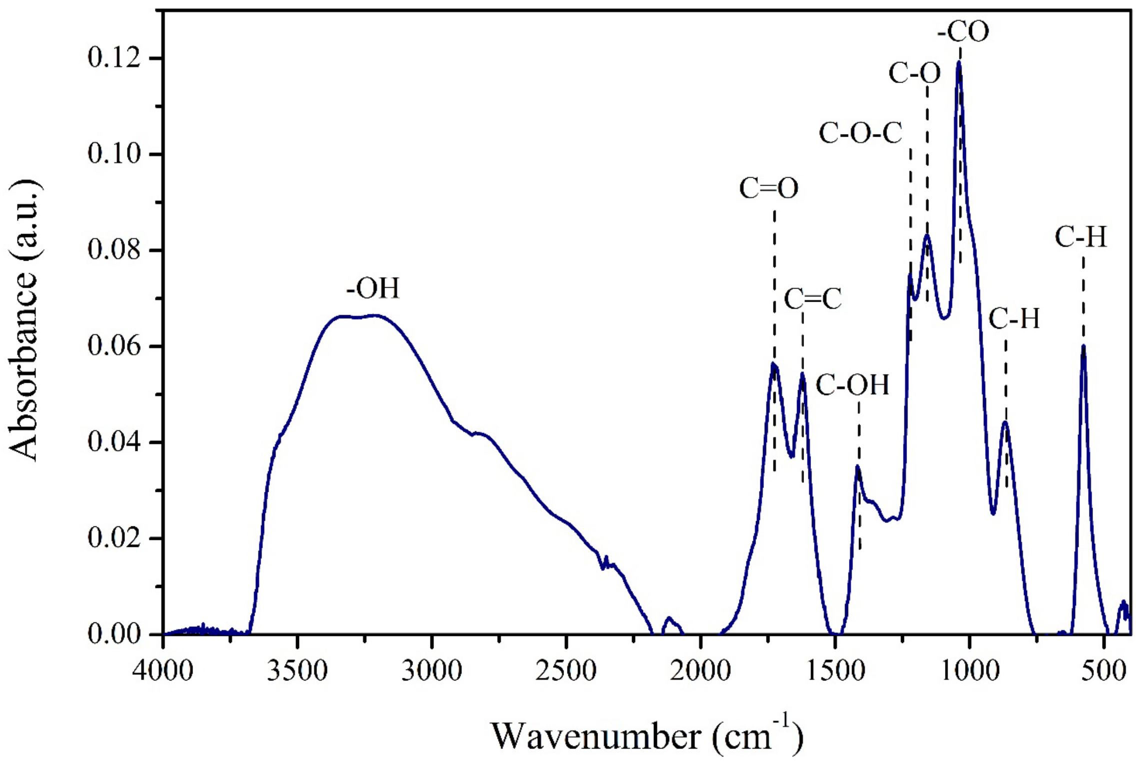

Figure S1 FTIR spectra of graphite oxide

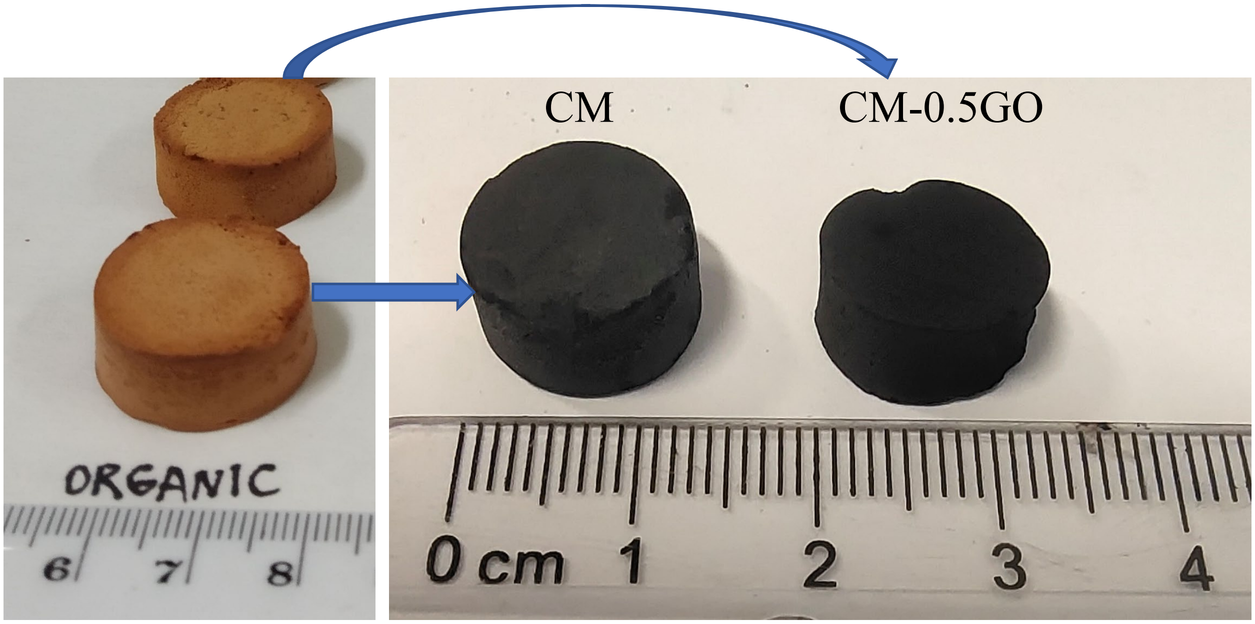

Figure S2 Pictures of prepared CM samples

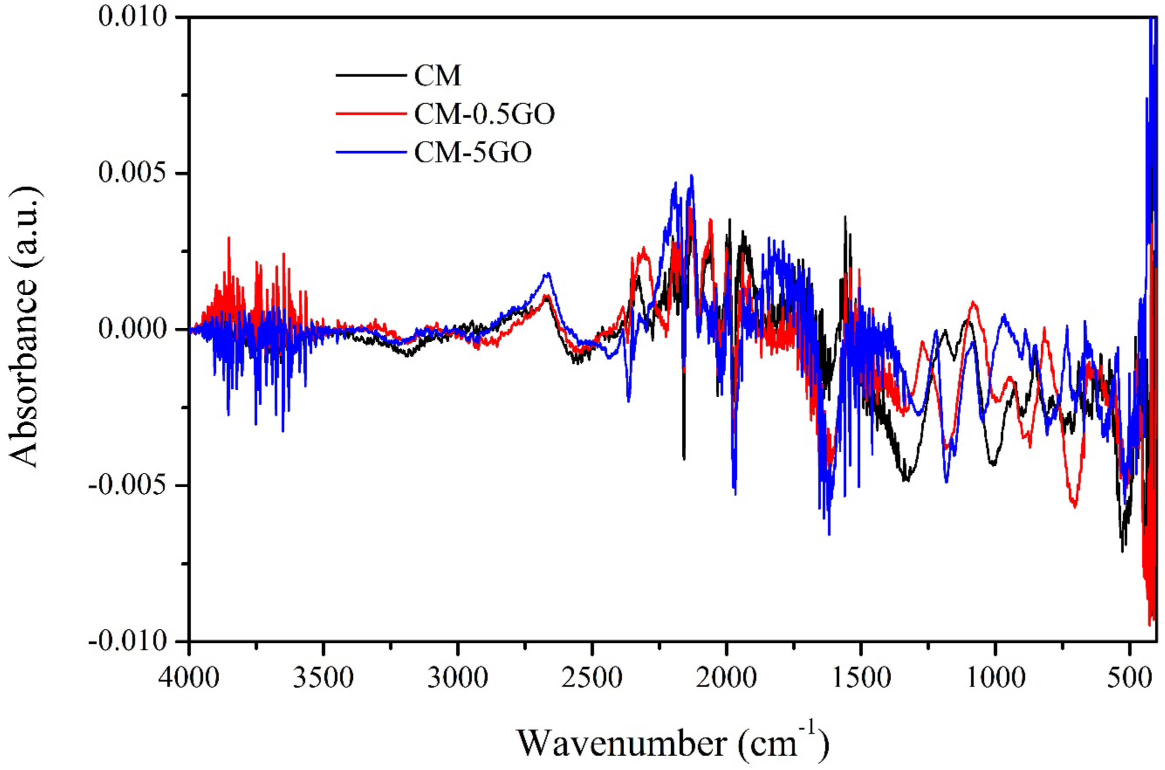

Figure S3 FTIR spectra of CM samples

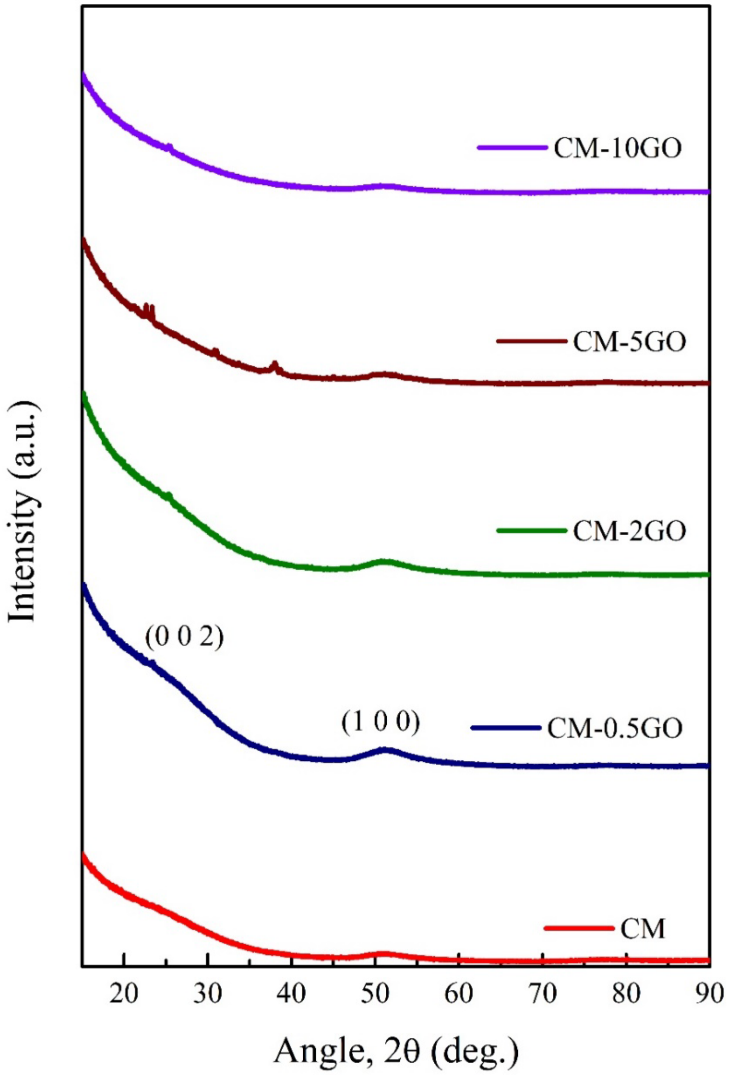

Figure S4 XRD spectra of CM samples

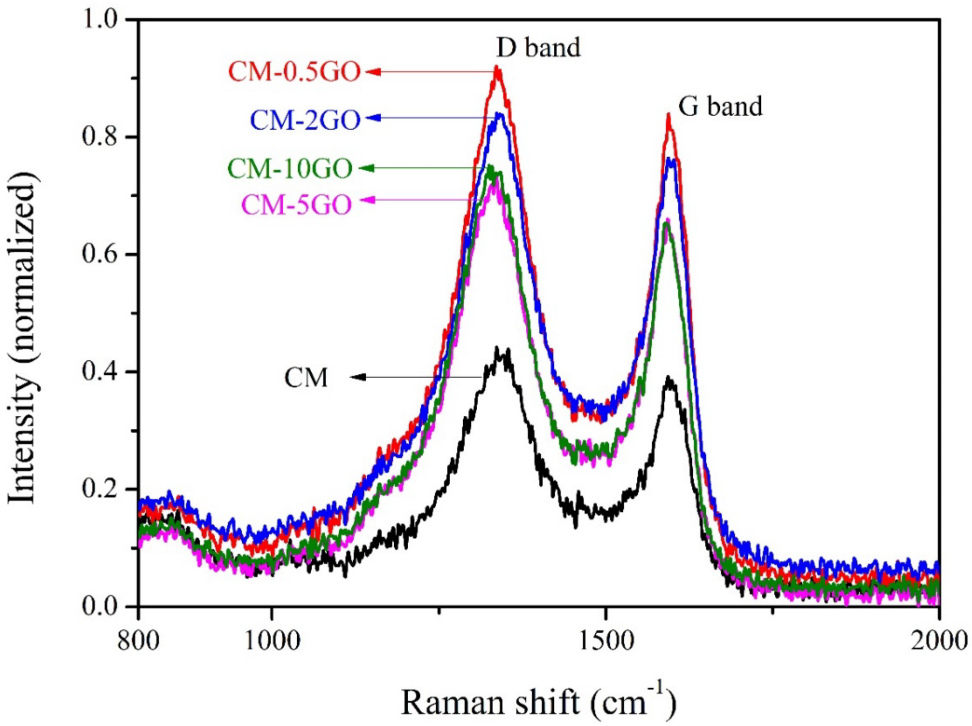

Figure S5 Raman spectra of CM samples

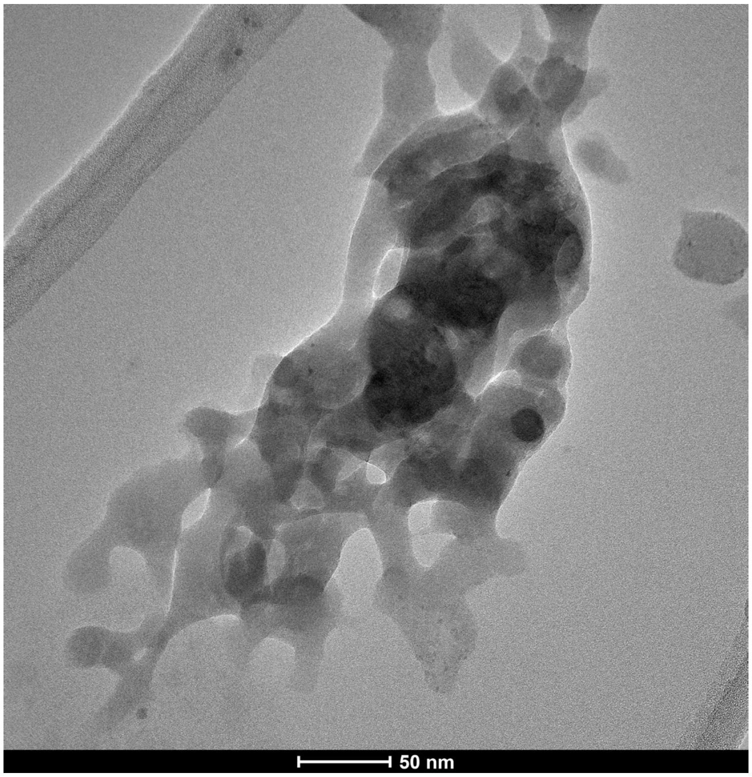

Figure S6 TEM image of CM-2GO

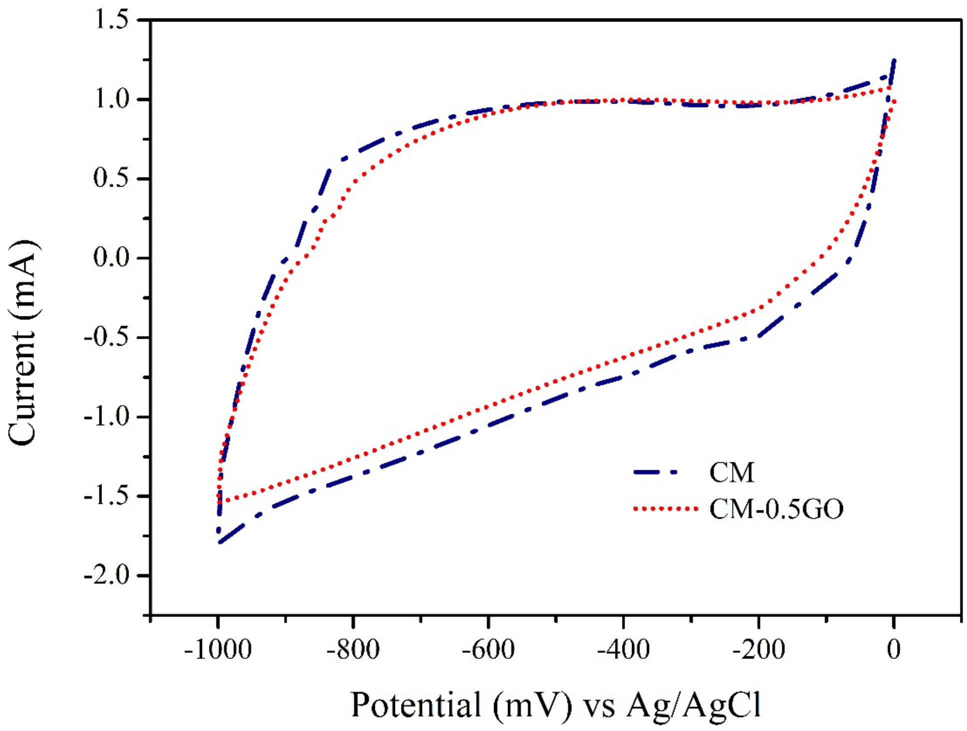

Figure S7 CV plot in a three-electrode cell at 50 mV s^{-1}

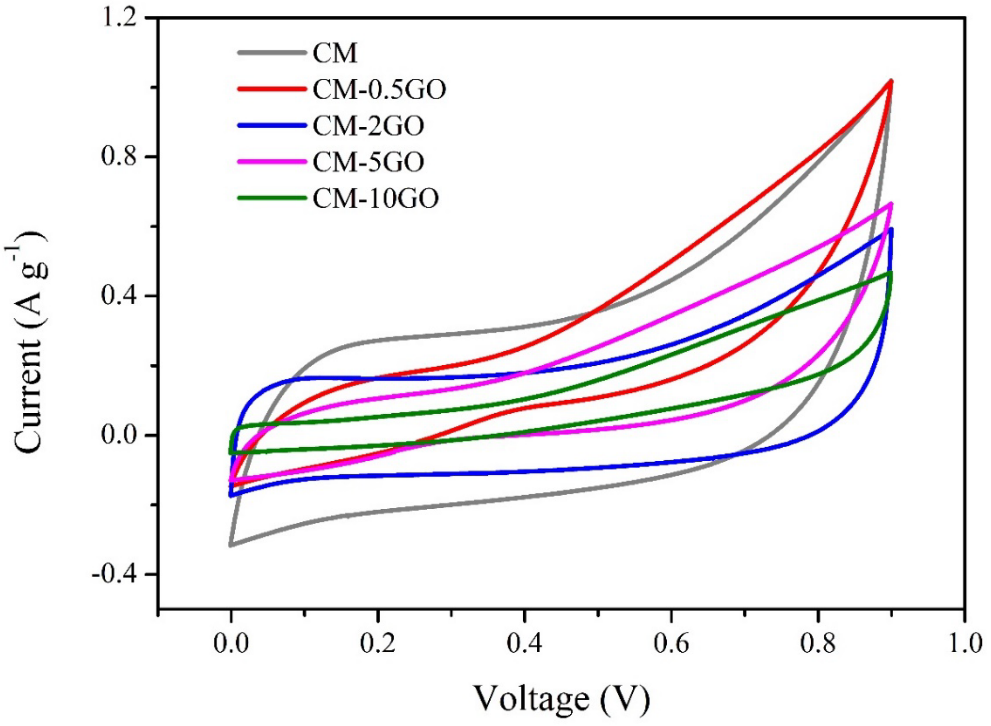

Figure S8 CV plot in a two-electrode cell at 5 mV s^{-1}

Table S1 Comparison of the electrochemical performance of CM electrodes with recently reported carbon electrodes from resorcinol-formaldehyde precursors under a two-electrode symmetric cell in an aqueous electrolyte

**Type of Carbon electrode**	**Electrolyte**	**Operating voltage (V)**	**Specific energy (Wh kg^{-1})**	**Corresponding specific power (W kg^{-1})**	**Ref. listed in the Ragone plot**
KOH activated carbon xerogel	2 M KOH	0 – 0.8 V	10	400	[17]
N-doped Yolk-shell carbon sphere	6 M KOH	0 – 1 V	14.33	499.9	[37]
1-D structured carbon	6 M KOH	0 – 1 V	8.77	250	[38]
Carbon xerogel modified using GO and ZnO template	0.5 M H_2SO_4	0 – 0.8 V	14.8	200.7	[39]
N-doped carbon-carbon nano-onion composite	0.1 M KOH	0 – 0.7 V	5	430	[40]
Tank-like shaped carbon sphere	6 M KOH	0 – 1 V	6.68	484	[41]
N-doped hollow carbon nanotubes	6 M KOH	0 – 1 V	12.5	247.9	[42]
Carbon sphere	6 M KOH	0 – 0.8 V	4.5	800	[43]
CM	2 M KOH	0 – 0.9 V	23.06	1203	This work
